# New Medical Building for Baltimore

**Published:** 1881-07

**Authors:** 


New Medical Building for Baltimore.—The project of erecting a new medical hall for the accommodation of the medical societies and associations of Baltimore, and the proper disposition and display of the large and valuable library belonging to the State society, is fast assuming shape and definiteness. And if kept in the hands of the committee now in charge of its interests, must ere long reach a satisfactory consummation. Should the plan that is contemplated by the committee be eventually adopted, the new building will not only fulfil all the present and future wants of the medical fraternity of the City and State, for many years to come, but will be an architectural ornament to the city likewise. The projectors and the committee have our best wishes in the premises.

